# A Model of the Statistical Power of Comparative Genome Sequence Analysis

**DOI:** 10.1371/journal.pbio.0030010

**Published:** 2005-01-04

**Authors:** Sean R Eddy

**Affiliations:** **1**Howard Hughes Medical Institute and Department of Genetics, Washington University School of MedicineSaint Louis, MissouriUnited States of America; Pennsylvania State UniversityUnited States of America

## Abstract

Comparative genome sequence analysis is powerful, but sequencing genomes is expensive. It is desirable to be able to predict how many genomes are needed for comparative genomics, and at what evolutionary distances. Here I describe a simple mathematical model for the common problem of identifying conserved sequences. The model leads to some useful rules of thumb. For a given evolutionary distance, the number of comparative genomes needed for a constant level of statistical stringency in identifying conserved regions scales inversely with the size of the conserved feature to be detected. At short evolutionary distances, the number of comparative genomes required also scales inversely with distance. These scaling behaviors provide some intuition for future comparative genome sequencing needs, such as the proposed use of “phylogenetic shadowing” methods using closely related comparative genomes, and the feasibility of high-resolution detection of small conserved features.

## Abstract 2

The mathematical model presented in this work will help to inform comparative genomics strategies for identifying conserved DNA sequences

## Introduction

Comparative genome sequence analysis is a powerful means of identifying functional DNA sequences by their evolutionary conservation [1,2,3]. It will be instrumental for achieving the goal of the Human Genome Project to comprehensively identify functional elements in the human genome [4]. How many comparative genome sequences do we need? Where is the point of diminishing returns, after which sequencing another koala or bat does not contribute significant information to human genome analysis? Since sequencing is expensive and capacity remains limited, one would like to address this issue as rigorously as possible.

Empirical evaluations of candidate comparative genomes have become important in allocating sequencing resources. Pilot sequencing and analysis in *Saccharomyces* and *Drosophila* species were done to choose appropriate species for comparative genome sequencing [5,6]. A pilot sequencing effort is underway for a number of mammalian genomes to evaluate their utility for human genome analysis [4]. Given the complexity of genomes, empirical studies are necessary. However, one would also like to complement this with higher-level, general insights that are independent of the details of particular analysis programs, organisms, and genomic features.

Cooper et al. proposed a mathematical model of one important type of comparative genome analysis [7]. They framed a question amenable to quantitative modeling: how many comparative genomes, and at what distances, are required to detect that an individual base in a target genome is “neutral” (inferred to be evolving at the neutral rate) as opposed to “conserved” (inferred to be under purifying selection)? Their model infers a nucleotide site to be conserved if it is 100% identical to homologous sites in *N* comparative genomes. The key parameters are the independent branch lengths *(d_i_)* contributed to a phylogeny by each new comparative genome *(i),* measured in neutral substitutions per site. More neutral evolutionary distance makes it more likely that neutral sites will have one or more substitutions in the alignment. Analytical strength increases as a function of the total neutral branch length in the phylogeny (Σ*_i_d_i_* ), because the probability that a neutral site has no changes in any branch of the phylogeny (and thus would be misclassified as conserved) is taken to be approximately *e* ^−Σ*_i_d_i_*^ . Based on the model, they concluded that 5.0 neutral substitutions/site of total branch length (about 10–20 well-chosen mammalian genomes) would approach “single nucleotide resolution” for human genome analysis, with a false positive probability (FP) of less than *e* ^−5.0^ per invariant site.

This model has some limitations that seem serious enough to question the proposed target of 10–20 mammalian genomes. Most importantly, it assumes that conserved sites are invariant. Few conserved features are absolutely invariant. If invariance is required to infer conservation, the fraction of truly conserved sites that are wrongly inferred to be neutral (because a substitution is seen in one of the comparative genomes) asymptotically approaches one as the number of comparative genomes or their evolutionary distance increases. We want to consider not just our FP, but our statistical power—our ability to successfully detect features that are conserved.

Additionally, single nucleotide resolution may not be the most relevant goal. It is useful to consider single nucleotide resolution as an ultimate limit on comparative analyses—one can imagine plausible analyses of single bases, and certainly individual codons—but we are mostly concerned with identifying conserved features of greater length, such as exons or transcription factor binding sites.

Nonetheless, the level of abstraction introduced by Cooper et al. is attractive. There is a need for better intuitions for planning comparative genome sequencing. How many more comparative genomes are needed as one looks for smaller and smaller conserved features—from exons to regulatory sites to single codons or even single nucleotides? How many more genomes are needed as one uses more and more closely related comparative genomes, in order to improve the chances that homologous lineage-specific features are found and correctly aligned [8,9]? Precise answers will be elusive, because genome biology is complex, but perhaps there are rough, useful scaling relationships amongst comparative genome number, evolutionary distance, and feature size. To explore this, I have extended the ideas introduced by Cooper et al. and developed an abstract model that seems to capture the essential flavor of comparative genome analysis.

## Results/Discussion

### Description of the Model

A “feature” is a sequence of *L* nucleotide sites in the target genome. We assume we have a correct, ungapped multiple sequence alignment of this sequence to *N* homologous features from *N* additional comparative genomes, and that the *L* sites are independent.

In the *NL* nucleotides in the aligned comparative sequences, we count how many changes are observed relative to the target feature sequence; call this *c*. If *c* is greater than some threshold *C,* we infer the feature is evolving at the neutral rate. If, on the other hand, *c* is less than or equal to *C,* we infer the feature is conserved.

We assume that each comparative genome is independently related to the target genome by a branch length of *D* neutral substitutions per site, that is, a uniform star topology, with the target at the root, and equal length branches to the comparative genomes at the leaves. A uniform star topology allows us to model how evolutionary distance affects comparative analysis at an abstract level, as a single variable *D,* independent of the details of real phylogenies. The biologically unrealistic placement of the known target at the root simplifies the mathematics, and does not significantly affect the results compared to making the more realistic assumption of an unknown ancestor at the root of a tree with *N* + 1 leaves, including the target.

We assume that the only difference between conserved features and neutral features is that conserved features evolve more slowly, by a relative rate coefficient *ω*. A conserved site accumulates an average of *ωD* substitutions, whereas a neutral site accumulates an average of *D* substitutions. *ω =* 0 for an absolutely conserved feature; *ω =* 1 for a neutrally evolving feature. At short evolutionary distances, we expect about *c = DNL* changes in neutral features, and *c* = *ωDNL* changes in conserved features, with binomial densities for *P*(*c*) around those values.

To model the probability that two nucleotides diverged by *D* or *ωD* substitutions will be observed to be identical (to deal with multiple substitutions at one site), we assume a Jukes-Cantor process in which all types of base substitution occur at the same rate [10]. Under a Jukes-Cantor model, the probability that two sites that have diverged by *D* substitution events are identical is 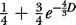, which approaches 25% at infinite divergence.

Given these assumptions, the FP in a comparative analysis (the probability that we erroneously infer that a neutral feature is conserved) is the probability that a neutral feature happens to have *C* or fewer observed changes (a cumulative binomial distribution):



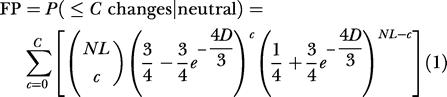



and the false negative probability (FN; the probability that we erroneously infer that a conserved feature is neutral) is the probability that a conserved feature happens to have more than *C* observed changes:



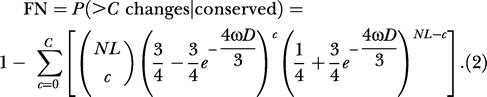



The model therefore depends on four parameters: the size of the conserved feature, *L,* the relative rate of evolution of the conserved feature, *ω,* the number of comparative genomes, *N,* and the neutral distance of the comparative genomes from the target genome, *D*. The threshold *C* is usually not an input parameter (except in the special case of invariance; *C* = 0). Rather, we find the minimum genome number *N* (or feature size *L*) at which there exists any cutoff *C* that can satisfy specified FN and FP thresholds.

The Cooper et al. model is essentially a special case where *L* = 1 (single nucleotide resolution), *ω =* 0 (conserved sites are always invariant), *C =* 0 (only invariant sites are inferred to be conserved), and FN = 0 by definition (if all conserved sites are invariant, and all invariant sites are inferred to be conserved, then all conserved sites are detected). Also, instead of using an evolutionary model to account for multiple substitutions at one site (saturation), Cooper et al. make a Poisson assumption that the probability of observing no change at a comparative site is *e^−D^,* which is only valid for small *D*.

The model discriminates features based on their relative rate of evolution. The same equations could be used to detect features evolving faster than the neutral rate (positively selected features), or to detect highly conserved features on a background of less strongly conserved sequence, as, for instance, transcription factor binding sites in an upstream region often appear [11,12]. For simplicity, I will only talk about discriminating “conserved” from “neutral” features here.

### Reasonable Parameter Values

The feature length *L* and conservation coefficient *ω* abstractly model the type of feature one is looking for. I use *L* = 50, *L* = 8, and *L* =1 as examples of detecting small coding exons, transcription factor binding sites, and single nucleotides, respectively, solely by sequence conservation. On average, conserved exons and regulatory sites appear to evolve about 2- to 7-fold slower than neutral sequences (*ω =* 0.5–0.15) [7,8,13,14,15]. I use 5-fold slower (*ω =* 0.2) in most cases discussed below. Typically, one doesn't know *L* or *ω* when looking for novel features. These two parameters behave as bounds: if one can detect a specified feature, larger and/or more conserved features are also detected.

The model's single distance parameter, *D,* abstractly represents the independent neutral branch length contributed by each comparative genome [7]. In a phylogenetic tree of the target with *N* > 1 comparative genomes that are as independent from each other as possible, we can roughly consider the independent branch length contributed by each comparative genome to be one-half its pairwise distance to the target genome, because in a real tree (with unknown common ancestors, as opposed to placing the target at the root of a uniform star topology) all comparative genomes share at least one branch leading to the target. Thus the figures highlight *D* = 0.03, 0.19, and 0.31 as “baboon-like,” “dog-like,” and “mouse-like” distances from human, 50% of one set of pairwise neutral distance estimates of 0.06, 0.38, and 0.62, respectively, arbitrarily chosen from the literature [7]. These labels are solely to give some intuition for what the model's *D* parameter means. The correspondence between *D* and real branch lengths is crude. Real neutral distance estimates are a subject of substantial (up to about 2-fold) uncertainty in the literature, and there are regional variations and strong context effects on neutral substitution rates in mammalian genomes [16,17]. More importantly, the model's uniform star topology, though it allows a high-level analysis in terms of just two parameters, *D* and *N,* makes direct comparison to real phylogenies difficult. Large numbers of equidistant, independently evolved mammalian genomes do not occur in reality. Real genomes are not independent, and will generally contribute an independent neutral branch length of less than one-half of their pairwise distance to the target genome.

**Figure 1:**
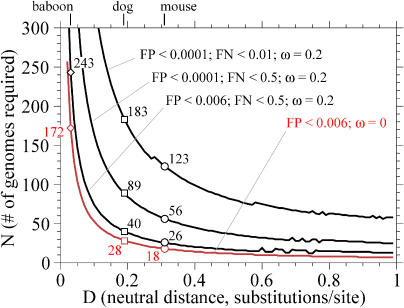
Number of Genomes Required for Single Nucleotide Resolution The red line plots genome number required for identifying invariant sites (*ω* = 0) with a FP of 0.006, essentially corresponding to the Cooper model [7]. Black lines show three more parameter sets: identifying 50% (FN < 0.5) of conserved sites evolving 5-fold slower than neutral (*ω* = 0.2) with FP < 0.006, doing likewise but with a more-stringent FP of 0.0001, and identifying 99% of conserved sites instead of just half of them. Values of *N* at baboon-like, dog-like, and mouse-like neutral distances are indicated with diamonds, squares, and circles, respectively. Jaggedness of the lines here and in subsequent figures is an artifact of using discrete *N, L,* and cutoff threshold *C* to satisfy continuous FP and FN thresholds.

**Figure 2:**
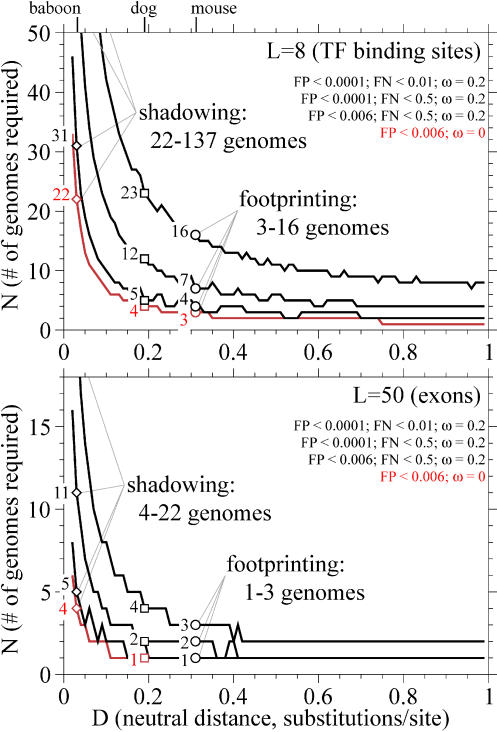
Number of Genomes Required for 8-nt or 50-nt Resolution Top: identifying 8-nt conserved features (“transcription factor binding sites”; *L =* 8); bottom: identifying 50-nt conserved features (“exons”; *L =* 50). Parameter settings are indicated at top right, in same order as the plotted lines. The parameters are the same as those used in Figure 1.

Critically, the model assumes that homologous features are present, correctly detected, and correctly aligned. In reality, with increasing evolutionary distance, features can be gained, lost, or transposed [14,18,19,20,21], the ability to detect homology by significant sequence similarity decreases, and alignments become less reliable [22]. The frequency of effects like loss, gain, and transposition depend on the biology of particular types of features, so departures from the model's “alignment assumptions” are difficult to model abstractly. However, minimally, we can posit a maximum neutral distance, *D*_max_, beyond which the alignment assumptions will not hold, based just on the ability of alignment programs to recognize and align homologous DNA sequences. Roughly speaking, reliability of DNA sequence alignments begins to break down at about 70% pairwise identity. For alignments of conserved features evolving 5-fold slower than neutral, this suggests *D*_max_ ∼ 0.15/0.2 = 0.75; Figures 1 and 2 show results out a little further, to *D*_max_ = 1.0.

Two different FP settings are used as illustrative examples: 0.006 (the *e* ^−5^ threshold used by Cooper et al. [7]) and the more stringent 10^−4^. For consistency, the same two FP thresholds are used to illustrate scaling behaviors for all three feature sizes (*L* = 1, *L* = 8, and *L* = 50). However, for a real analysis, one wants to consider the appropriate choice of FP carefully. In a genome sequence of length *M,* the total number of false positive feature predictions in all overlapping possible windows of length *L* is *M − L +* 1, multiplied by FP per feature. In most analyses, we would probably merge overlapping predicted features into a single predicted conserved region, resulting in a lower number of false positive regions in a genome. This overlap correction (from the number of false features to the number of false regions) depends on the parameters, but for the parameters in Figures 1 and 2 it varies from 1.5- to 2-fold less for *L* = 8 sites and 4- to 8-fold less for *L* = 50 sites, based on simulations. Thus, for example, FP = 10^−4^ corresponds to one false positive feature per 10 kb, and (for the parameters here) somewhere between one false positive conserved region per 20–100 kb, depending on the feature. For “small exon” detection, this means 40,000–300,000 false region/feature predictions in the 3-Gb human genome; for “transcription factor binding sites,” this means one false positive feature or region per 10–20 kb. FP = 10^−4^ therefore seems a reasonable stringency for *L =* 8 or *L =* 50 feature analyses. If one carried out a single nucleotide resolution analysis on a genome-wide scale, FP = 10^−4^ would mean that 99.8% of the predictions for conserved bases in the 3-Gb human genome would be correct, assuming about 5% of the bases are truly conserved and detected with high sensitivity. However, it is likely that one would actually carry out single nucleotide resolution analyses on a subset of conserved features that had already been identified (exons, for example), so a less stringent FP might be required. The setting of FP = 0.006 might therefore be more appropriate for evaluating single nucleotide resolution, where FP is closer to the traditional statistical choices of a 0.01 or 0.05 significance level.

### Single Nucleotide Resolution Requires Many Genomes

The Cooper model concluded that for invariant conserved sites, sequencing comparative genomes to achieve a total branch length of five neutral substitutions per site would give single nucleotide resolution, with a FP of *e* ^−5^ (0.006) [7]. Under my model, detection of invariant nucleotides takes about 17 genomes at mouse-like distances, essentially as predicted by Cooper et al. (Figure 1).

However, the picture changes when one considers comprehensive detection of features that are conserved but not invariant (Figure 1). To detect 50% of sites evolving 5-fold slower than neutral, we need 25 comparative genomes at mouse-like distances at the same (arbitrary) false positive threshold of less than 0.006. For a comprehensive screen that would detect 99% of conserved single nucleotides with a FP of less than one per 10 kb, the model predicts about 120 comparative genomes at mouse-like distances are needed.

### Detectable Feature Size Scales Inversely with Genome Number

The large genome numbers in Figure 1 might appear to conflict with the known power of comparing just two genomes, such as human and mouse. This is because recognizing conserved sequences is easier than recognizing conserved single nucleotides; the size of the conserved feature matters.

Figure 2 shows how many genomes are required to detect small features like transcription factor binding sites (*L* ∼ 8) or larger features like short coding exons (*L* ∼ 50). One genome at about human/mouse distance is sufficient for reasonable strength in coding exon detection. For a range of reasonable sensitivity and specificity stringencies, three to 15 genomes at human/mouse distance are sufficient for detecting transcription factor binding sites.

There is a general, intuitive explanation for this. The strength of an analysis will depend on the difference in the expected number of substitutions in neutral features versus conserved features. This difference will be proportional to *NL,* the total number of aligned sites. Thus, for a constant stringency, the required number of comparative genomes is expected to scale inversely with the size of the feature to be detected (*N* ∝ 1*/L*): to detect conserved features ten times smaller, it takes ten times as many comparative genomes. (This scaling behavior is seen directly later.)

### No Clear Optimum for Evolutionary Distance, but Close Distances Disfavored

Figures 1 and 2 show two other notable behaviors. First, there is no sharp optimum for the neutral distance *D*. The number of genomes required is relatively flat for a wide range, from about 0.4 to well beyond 1.0. Within a broad range, the exact choice of one comparative genome versus another has little impact.

**Figure 3:**
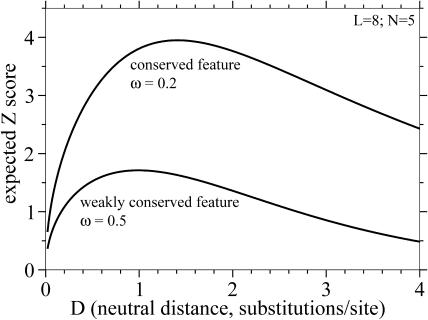
A Measure of Statistical Strength As a Function of Neutral Evolutionary Distance One convenient threshold-independent measure of the strength of a comparative analysis is an expected *Z* score, the expected difference Δ*c* in the number of substitutions in a neutral feature alignment versus a conserved feature alignment, normalized to units of standard deviations. *E*(*Z*) is readily calculated for the binomial distribution: 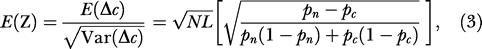 where *p_n_* and *p_c_* are the probabilities of observing a change at one aligned comparative nucleotide according to the Jukes-Cantor equation. The plots here are for *N* = 5 and *L* = 8. The shape of the curve is independent of *N* and *L,* while the absolute magnitude of *Z* scales as √*NL* . The x-axis is shown from *D =* 0 to *D* = 4, beyond the more realistic range of Figures 1 and 2, to show the mathematically optimum *D* if homologous conserved features were present, recognized, and accurately aligned at any *D*.

This is shown more directly in Figure 3, in which a measure of overall statistical strength is plotted against neutral distance over an unrealistically long range of *D,* out to 4.0 substitutions/site. For conserved features evolving 5-fold slower than neutrality, assuming that alignment assumptions hold, the optimum distance according to the model is about 1.4 neutral substitutions/site, four to five times the mouse-like distance. However, for many kinds of features, at such long evolutionary distances the alignment assumptions are likely to break down. Because the mathematically optimal distance for discriminating idealized conserved and neutral features lies outside the range where the alignment assumptions are likely to hold, it may not be particularly meaningful to imagine a uniquely optimal choice of evolutionary distance for comparative genome analysis; optimal choices will be problem-dependent. (This is not surprising, of course, but perhaps useful to see clearly in a simple model.)

The second behavior worth noting in Figures 1 and 2 is that at close evolutionary distances, the necessary number of comparative genomes needed ramps up steeply. For instance, at human/baboon distances of 0.03, achieving equivalent statistical strength requires about seven times as many comparative genomes as when using human/mouse distances (see Figure 2).

There is another general intuition behind these results. For *D* ≪ 1, the expected number of substitutions is *DNL* in a neutral feature and *ωDNL* in a conserved feature. So, for a constant statistical stringency, the number of genomes required will scale inversely with evolutionary distance, when the distance is small. At larger distances, this scaling ceases as the number of observed changes saturates.

The strong scaling of *N* at small distances *D* has implications for the use of “phylogenetic shadowing” using closely related genomes [8,9]. It is clear that the use of closely related genomes is advantageous in several ways: alignments are more accurate, one can accurately align a surrounding neutral region to detect small embedded conserved regions, and homologous features are more likely to be present (for instance, primate-specific features in human analyses). However, the model illustrates how these advantages are accompanied by a significant cost in statistical strength (see Figure 3). When using comparative species at short evolutionary distances, species choice matters a lot. Within primates, for example, divergence times from human vary about 10-fold (∼6 to ∼65 million years); if one aims to use “primate sequences” for human genome analysis, there is a large difference between using distant primates (lemurs or New World monkeys) versus close primates (great apes).

### Resolution and Stringency as a Function of Genome Number

**Figure 4:**
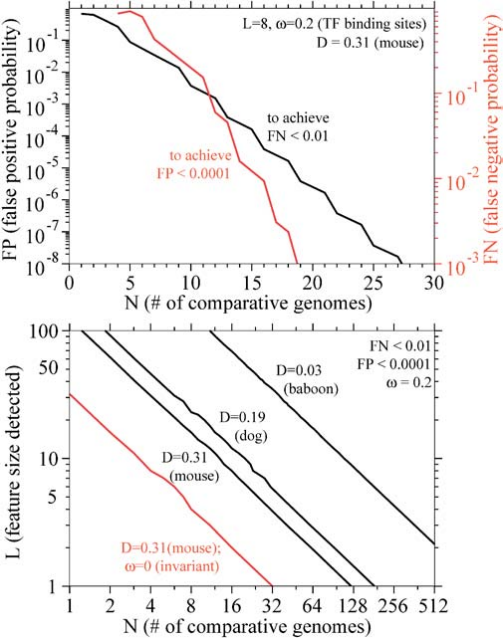
Increase in Stringency and Resolution with Increasing Genome Number Top: black line shows improvement in specificity (FP) for transcription factor (TF) binding site–like features (*L* = 8, *ω* = 0.2) as comparative genome number increases, for FN = 0.01 (99% of sites detected), and genomes of *D* = 0.31 (mouse/human-like distance). Red line shows improvement in sensitivity (FN) for the same parameters and a FP threshold of 0.0001. Shown as a log-linear plot to show the expected rough log(FP or FN) proportional to −*N* scaling. Bottom: resolution (size of detectable feature, *L*) as a function of comparative genome number, plotted on log-log axes to show the fit to the expected *L* ∝ 1/*N* scaling. All four lines assume goals of FN < 0.01 and FP < 0.0001. Black lines are for identifying conserved features evolving 5-fold slower than neutral (*ω* = 0.2), using baboon-like (*D* = 0.03), dog-like (*D* = 0.19), or mouse-like (*D* = 0.31) genomes. Red line is for identifying invariant features with mouse-like genomes.

How much additional information does each new comparative genome sequence give us? The top panel in Figure 4 plots sensitivity and specificity as the number of comparative genomes increases, for an analysis of transcription factor binding site–like features. The scaling behavior is expected to be (roughly speaking) log(FP or FN) ∝ − *N*, based on the cumulative binomial expressions for FP and FN. That is, each additional genome reduces FP or FN by a roughly constant multiplier; for the parameters used here, every three or four more comparative genomes reduces FP by 10-fold. The bottom panel in Figure 4 plots resolution *L* as a function of *N,* showing the expected *L* ∝ 1/*N* scaling. Each doubling of the number of comparative genomes increases resolution about 2-fold.

### Good Agreement with More Realistic Simulations

The model's simplicity is useful. By just counting the number of substitutions in conserved versus neutral features, the reasons for the scaling behaviors are more intuitively obvious. However, the assumptions required for this level of simplicity are questionable. In real DNA sequences, the Jukes-Cantor model's simple assumptions are violated in many ways; transitions are more frequent than transversions, base composition is not uniform, and mutation rates show strong context dependence [17]. In a real analysis, we would use probabilistic methods to compare the log likelihood ratio (LLR) of a phylogenetic tree under competing hypotheses of two different rates [8,23,24], so we can deal with real phylogenies and different expected rates of substitutions at different bases.

The relative predicted scaling behaviors are unlikely to change under more realistic simulations. However, for the model to be useful as a rough guide for required genome number under different comparative analysis scenarios, at a minimum we want to know whether the absolute predicted numbers would be substantially different for features evolving under a more realistic evolutionary model, such as the Hasegawa-Kishino-Yano (HKY) model [25], which models nonuniform base composition and transition/tranversion rate bias, and if we analyzed those data with LLR statistics instead of simply counting substitutions.

Therefore, I performed the following computational simulation study. Synthetic “neutral” and “conserved” feature alignments were generated using two HKY models that differed in evolutionary rate by a factor of *ω*. The rates in the HKY models were parameterized with an AT-biased base composition of 33% A, 17% C, 17% G, and 33% T, and a biased transition/transversion rate ratio of 4.0. A feature alignment was simulated by choosing a random *L*-mer (using the specified base composition) as the target feature, then generating *N* homologous features from it with substitutions according to an HKY conditional substitution matrix at distance *D*. For each dataset, 10^3^ conserved feature alignments and 10^6^ neutral feature alignments were generated. These alignments were then scored under the two HKY models and ranked by LLR score. This was repeated for increasing *N* until an LLR score threshold existed that could satisfy the chosen FP and FN thresholds. I then reproduced the analyses in Figures 1 and 2 using the HKY/LLR simulation for the 27 highlighted points with *ω* = 0.2. That is, for the 27 combinations of *D* = 0.03, 0.19, or 0.31; *L* = 1, 8, or 50; and (FP, FN) = (0.0001, 0.01), (0.0001, 0.5), or (0.006, 0.5), I determined the minimum number of genomes required to achieve the chosen thresholds.

This analysis showed that the predictions of the simple model's equations and the results of the HKY/LLR simulations are in close agreement. The maximum deviation was 15%. For example, for the [*D* = 0.19, *L* = 1] points where the model predicts needing *N* = 183, 89, and 40 for the different values of FP and FN, the HKY/LLR simulation predicts needing *N* = 210, 80, and 35; for the [*D* = 0.19, *L* = 8] points, the model predicts *N* = 23, 12, and 5, and the simulation predicts *N* = 24, 11, and 5; and for the [*D* = 0.19, *L* = 50] points the model and simulation both predict *N* = 4, 2, and 1.

More significant discrepancies appear at larger distances. A simple Jukes-Cantor model has only one substitution rate, so all types of substitutions saturate equally fast. In an HKY model, some substitution rates are faster than others. Intuitively, one expects an HKY model to be able to extract information from slower, less quickly saturated substitutions at longer distances, resulting in more discrimination at large *D* than the simple model predicts. This effect appears at distances of *D* > 2.0–3.0 or so: for instance, for [*ω* = 0.2, *L* = 8] features, to achieve FP < 0.0001 and FN < 0.01 for distances of *D* = 1, 2, 3, 4, 5, and 10, the simple model predicts needing *N* = 8, 8, 11, 17, 26, and 335 genomes, respectively, whereas HKY/LLR simulations predict needing *N* = 8, 9, 9, 13, 17, and 82 genomes. Thus, the simple model's approximation breaks down somewhat at larger distances, beyond the *D* < 1 range that is considered here to be reasonable for comparative genomics.

Additionally, nonuniform base composition causes some composition-dependent spreading around the mean *N* that is not predicted by the simple model. For instance, GC-rich features are more easily detected than AT-rich features when substitution rates are biased towards high AT composition. Additional HKY/LLR simulations, using the same HKY matrices as above but specifically looking at poly-A features versus poly-C target features, show this effect; for instance, for [*ω* = 0.2, *L* = 8] features at *D* = 0.19, to achieve FP < 0.0001 and FN < 0.01, we need at least *N =* 24 genomes to detect features on average, but specifically we need *N =* 19 for poly-C/G features and *N* = 29 for poly-A/T features.

### Reasonable Agreement with Available Data

One also wants to see that the model's predictions do not disagree with published results, at least to the extent that it is possible to crudely compare real phylogenies to the abstracted uniform star topology of the model. Three examples follow.

Cooper et al. estimated that the mouse and rat genomes suffice for about 50-nt resolution of human conserved features [26]. The independent branch lengths to human, mouse, and rat are roughly 0.3, 0.3, and 0.1 neutral substitutions/site; the rat is close to the mouse, so this situation is difficult to fit with a single *D*. However, using either *N* = 1 and *D* = 0.6 (pairwise comparison to one rodent using one full pairwise distance for *D*), or *N* =2 and *D* = 0.23 (approximating *D* as an average of three independent branch lengths), or *N* = 2 and *D* = 0.35 (approximating *D* as one-half the average pairwise distances from human to mouse and rat), the model predicts that 90% of 50-nt features with *ω* = 0.2 can be detected with a reasonable FP of between 0.0003 and 10^−5^; but for features just half that size (*L* = 25), FP collapses to between 0.02 and 0.006 (one false prediction every 50–150 nt).

Boffelli et al., in introducing “phylogenetic shadowing,” used 13–17 primate sequences with a total independent branch length of about 0.6 neutral substitutions/site to analyze conserved sequences smoothed in 50-nt windows, and conserved regions down to 40–70 nt were detected effectively [8]. The model predicts that for *N* = 15 and *D* = 0.04 (average independent branch length of 0.6/15), one can detect 90% of 50-nt, *ω* = 0.2 features with a FP of 10^−5^; but for 25-nt features, FP collapses to 0.003 (one false prediction per 300 bp).

Kellis et al. and Cliften et al. reported comparative analyses to identify transcription factor binding sites in Saccharomyces cerevisiae using alignments of intergenic regions to three comparative *Saccharomyces* genomes, with a total independent branch length of about 0.8–0.9 [11,12]. For *N* = 3 and *D* = 0.27–0.30 (average independent branch length of 0.8/3 to 0.9/3), the model predicts that binding site–like features (*L* = 8, *ω* = 0.2) would be detected with a FP of 0.001–0.002 (about one false prediction per upstream region) and a sensitivity of about 25%, suggesting that these data are barely sufficient to identify individual short conserved features. Indeed, though both research groups showed examples of highly conserved individual sites, both groups analyzed their data primarily at the level of detecting motif consenses, rather than attempting to detect individual features genome-wide. That is, they required that the same motif be found conserved in multiple places upstream of multiple genes. This is a data aggregation strategy, multiplying the effective *L* by the number of copies of the feature. In this way, even when only a fraction of individual features are identified, the existence of a conserved consensus motif may be inferred from the average conservation of the aggregated data.

### Limitations on the Generality of These Conclusions

The model assumes a pure, brute force detection of individual conserved features by comparative analysis. For many particular problems, one can leverage additional information and reduce the number of comparative genomes needed. Data aggregation strategies are one example (for instance, detecting that a particular consensus motif is conserved more often than expected, averaged across all individual occurrences [27]). Another strategy is to combine sequence conservation data with other experimental data (for instance, using microarray data to detect that a marginally conserved motif is also statistically associated with a coordinately regulated set of genes [28]).

Some features are not just conserved, but also show informative patterns of substitution, insertion, and deletion, so we can gain power by using feature-specific evolutionary models instead of a general conservation screen. For instance, coding regions predominately show substitutions in wobble positions and strong selection against insertions/deletions, and those insertions/deletions that remain will generally preserve frame [11]. Conserved structural RNAs reveal their basepaired secondary structure interactions by compensatory basepair mutations [29]. In such analyses it becomes important to see enough evolutionary events to distinguish one kind of conserved feature from other kinds of conserved features, not just to discriminate conserved from neutral. Because different conserved features evolve at different rates, one would generally want to have a range of comparative genomes at different distances, so that for any given conserved feature with its particular relative rate of evolution, one can find alignments in a “sweet spot” with the right amount of divergence.

Finally, there are other important uses of comparative genomics in addition to DNA sequence analysis of conserved elements. For example, evolutionary/developmental studies choose species based on phylogenetic position, and population genetics studies choose multiple individuals within the same species.

### Concluding Remarks

The principal results here are two inverse scaling behaviors that provide useful intuitions for planning comparative genome sequencing. All other things being constant, the required number of comparative genomes is inversely proportional to detectable feature size, and at small evolutionary distances, required genome number becomes inversely proportional to the neutral distance to the comparative genomes.

Neither behavior is entirely surprising; the contribution of an abstract model is to see them more clearly. Obviously, it takes more comparative genomes to recognize smaller features, though one may not have predicted a simple inverse relationship between *L* and *N*. And it is already common to use total independent neutral branch length as a measure of the strength of a comparative dataset [7,8,9,30], which implies an inverse relationship between genome number and evolutionary distance, a relationship made explicit in a simplified model where total independent branch length is *ND*.

The model also shows clearly that for two analysis scenarios—identification of small conserved features and the identification of lineage-specific conserved features in closely related genomes—it will be useful to obtain large numbers of comparative genome sequences. Since a small number of comparative genome sequences are already enabling powerful analyses, this may be surprising. Even for simple conservation analyses, we have not begun to exhaust the power of comparative genome analysis.

## Materials and Methods

The model was implemented in several ANSI C programs, which can be downloaded at http://www.genetics.wustl.edu/eddy/publications/Eddy05.

## Abbreviations

FP: false positive probability
FN: false negative probability
HKY: Hasegawa-Kishino-Yano
LLR: log likelihood ratio

## References

[pbio-0030010-b01] Hardison RC (2000). Conserved noncoding sequences are reliable guides to regulatory elements. Trends Genet.

[pbio-0030010-b02] Sidow A (2002). Sequence first. Ask questions later. Cell.

[pbio-0030010-b03] Hardison RC (2003). Comparative genomics. PLoS Biol.

[pbio-0030010-b04] Thomas JW, Touchman JW, Blakesley RW, Bouffard GG, Beckstrom-Sternberg SM (2003). Comparative analyses of multi-species sequences from targeted genomic regions. Nature.

[pbio-0030010-b05] Cliften PF, Hillier LW, Fulton L, Graves T, Miner T (2001). Surveying *Saccharomyces* genomes to identify functional elements by comparative DNA sequence analysis. Genome Res.

[pbio-0030010-b06] Bergman CM, Pfeiffer BD, Rincon-Limas DE, Hoskins RA, Gnirke A (2002). Assessing the impact of comparative genomic sequence data on the functional annotation of the *Drosophila* genome. Genome Biol.

[pbio-0030010-b07] Cooper GM, Brudno M, Green ED, Batzoglou S, Sidow A (2003). Quantitative estimates of sequence divergence for comparative analyses of mammalian genomes. Genome Res.

[pbio-0030010-b08] Boffelli D, McAuliffe J, Ovcharenko D, Lewis KD, Ovcharenko I (2003). Phylogenetic shadowing of primate sequences to find functional regions of the human genome. Science.

[pbio-0030010-b09] Boffelli D, Nobrega MA, Rubin EM (2004). Comparative genomics at the vertebrate extremes. Nat Rev Genet.

[pbio-0030010-b10] Jukes TH, Cantor CR, Munro HN (1969). Evolution of protein molecules. Mammalian protein metabolism.

[pbio-0030010-b11] Kellis M, Patterson N, Endrizzi M, Birren B, Lander ES (2003). Sequencing and comparison of yeast species to identify genes and regulatory elements. Nature.

[pbio-0030010-b12] Cliften P, Sudarsanam P, Desikan A, Fulton L, Fulton B (2003). Finding functional features in *Saccharomyces* genomes by phylogenetic footprinting. Science.

[pbio-0030010-b13] Makalowski W, Boguski MS (1998). Evolutionary parameters of the transcribed mammalian genome: An analysis of 2,820 orthologous rodent and human sequences. Proc Natl Acad Sci U S A.

[pbio-0030010-b14] Dermitzakis ET, Clark AG (2002). Evolution of transcription factor binding sites in mammalian gene regulatory regions: Conservation and turnover. Mol Biol Evol.

[pbio-0030010-b15] Moses AM, Chiang DY, Kellis M, Lander ES, Eisen MB (2003). Position specific variation in the rate of evolution in transcription factor binding sites. BMC Evol Biol.

[pbio-0030010-b16] Mouse Genome Sequencing Consortium (2002). Initial sequencing and comparative analysis of the mouse genome. Nature.

[pbio-0030010-b17] Hwang DG, Green P (2004). Bayesian Markov chain Monte Carlo sequence analysis reveals varying neutral substitution patterns in mammalian evolution. Proc Natl Acad Sci U S A.

[pbio-0030010-b18] Ludwig MZ, Patel NH, Kreitman M (1998). Functional analysis of eve stripe 2 enhancer evolution in *Drosophila* Rules governing conservation and change. Development.

[pbio-0030010-b19] Ludwig MZ, Bergman C, Patel NH, Kreitman M (2000). Evidence for stabilizing selection in a eukaryotic enhancer element. Nature.

[pbio-0030010-b20] Costas J, Casares F, Vieira J (2003). Turnover of binding sites for transcription factors involved in early *Drosophila* development. Gene.

[pbio-0030010-b21] Ludwig MZ (2002). Functional evolution of noncoding DNA. Curr Opin Genet Dev.

[pbio-0030010-b22] Pollard DA, Bergman CM, Stoye J, Celniker SE, Eisen MB (2004). Benchmarking tools for the alignment of functional noncoding DNA. BMC Bioinformatics.

[pbio-0030010-b23] Anisimova M, Bielawski JP, Yang Z (2002). Accuracy and power of Bayes prediction of amino acid sites under positive selection. Mol Biol Evol.

[pbio-0030010-b24] Yang Z (2002). Inference of selection from multiple species alignments. Curr Opin Genet Dev.

[pbio-0030010-b25] Hasegawa M, Kishino H, Yano T (1985). Dating of the human-ape splitting by a molecular clock of mitochondrial DNA. J Mol Evol.

[pbio-0030010-b26] Cooper GM, Brudno M, Stone EA, Dubchak I, Batzoglou S (2004). Characterization of evolutionary rates and constraints in three mammalian genomes. Genome Res.

[pbio-0030010-b27] Fairbrother WG, Yeo GW, Yeh R, Goldstein P, Mawson M (2004). RESCUE-ESE identifies candidate exonic splicing enhancers in vertebrate exons. Nucleic Acids Res.

[pbio-0030010-b28] Wang T, Stormo GD (2003). Combining phylogenetic data with co-regulated genes to identify regulatory motifs. Bioinformatics.

[pbio-0030010-b29] Rivas E, Eddy SR (2001). Noncoding RNA gene detection using comparative sequence analysis. BMC Bioinformatics.

[pbio-0030010-b30] Cooper GM, Sidow A (2003). Genomic regulatory regions: Insights from comparative sequence analysis. Curr Opin Genet Dev.

